# Effects of acteoside from *Cistanche tubulosa* on the plasma metabolome of cancer-related fatigue mice inoculated with colon cancer cells

**DOI:** 10.3389/fphar.2024.1370264

**Published:** 2025-01-13

**Authors:** Shilei Zhang, Fukai Gong, Jiali Liu, Shuping You, Tao Liu, Jianhua Yang, Junping Hu

**Affiliations:** ^1^ State Key Laboratory of Pathogenesis, Prevention and Treatment of High Incidence Diseases in Central Asia, Department of Central Laboratory, School of Public Health, Xinjiang Medical University, Urumqi, China; ^2^ Department of Pharmacy, People’s Hospital of Xinjiang Uygur Autonomous Region, Urumqi, China; ^3^ Department of Pharmacognosy, School of Pharmacy, Xinjiang Medical University, Urumqi, China; ^4^ Department of Basic Nursing, School of Nursing, Xinjiang Medical University, Urumqi, China; ^5^ Department of Toxicology, School of Public Health, Xinjiang Medical University, Urumqi, China; ^6^ Department of Pharmacy, State Key Laboratory of Pathogenesis, Prevention and Treatment of High Incidence Diseases in Central Asia, The First Affiliated Hospital of Xinjiang Medical University, Urumqi, China

**Keywords:** side effects of radiotherapy and chemotherapy, cancer-related fatigue, metabolomics, *Cistanche tubulosa*, acteoside

## Abstract

**Objective:**

To elucidate the metabolic mechanisms by which acteoside (ACT) isolated from *Cistanche tubulosa* alleviates cancer-related fatigue (CRF) in a murine model of colon cancer with cachexia.

**Methods:**

BALB/c mice inoculated with C26 colon cancer cells were treated with paclitaxel (PTX, 10 mg/kg) and ACT (100 mg/kg) alone or in combination for 21 days. Fatigue-associated behaviors, tumor inhibition rate, and skeletal muscle morphology assessed by hematoxylin-eosin (H&E) staining and electron microscopy were evaluated. Finally, liquid chromatography-mass spectrometry (LC/MS) was employed to investigate alterations in the plasma metabolic profile of tumor-bearing mice with CRF in response to ACT treatment, and the affinity between metabolite-associated proteins and ACT was verified by Surface plasmon resonance (SPR) assay.

**Results:**

Our study demonstrated the presence of CRF in the colon cancer mouse model, with the severity of fatigue increasing alongside tumor growth. Administration of ACT ameliorated both tumor burden and PTX-induced muscle fatigue-like behavior. LC/MS analysis identified a panel of differentially regulated metabolites, including trans-aconitine, citric acid, 3-coumaric acid, ephedrine, thymine, cytosine, indole-3-acetic acid, and pantothenol-9. These metabolites were primarily enriched in pathways associated with valine biosynthesis, tyrosine metabolism, tryptophan metabolism, and biosynthesis of pyridine alkaloids. Furthermore, several key enzymes, including CYP3A4, CYP19A1, CYP2E1, TNF, BCL-2, RYR2, and ATP2A1, were identified as potential targets underlying the anti-CRF effects of ACT.

**Conclusion:**

This study suggests that ACT derived from *C. tubulosa* harbors protective properties against cancer-related fatigue mediated by tumor cells.

## 1 Introduction

Colon cancer is a significant global health burden, ranking as the third most common malignancy and the second leading cause of cancer-related deaths after lung cancer (Araghi et al., 2019). Each year, an estimated one million people worldwide receive a colon cancer diagnosis, resulting in substantial patient suffering and placing a strain on healthcare systems (Sinicrope, 2022). Fortunately, advancements in diagnostic and treatment technologies, coupled with heightened public awareness about self-care, have led to a gradual decline in colon cancer mortality rates. Consequently, a growing population of individuals are either living with or have experience with colon cancer. This trend underscores the critical need to prioritize improving the quality of life for this expanding patient population.

CRF is a persistent and subjective sensation of tiredness, primarily manifesting as a lack of energy and general fatigue (Thong et al., 2020). Its occurrence is directly linked to cancer or its treatment, significantly impacting patients’ daily lives and overall quality of life. Studies have shown an escalating trend of CRF across various tumor types following initial treatment. The reported incidence rate ranges from 25% to 99% during treatment, rising to 65%–100% after chemotherapy and 82%–96% after radiotherapy. Notably, 30% of patients experience moderate to severe CRF (Bower, 2014). While successful treatment may alleviate CRF for some patients, follow-up studies reveal a persistence of fatigue in 25%–33% of patients, lasting for several years or exceeding 10 years post-diagnosis. This fatigue is not alleviated by adequate sleep or rest and may even disrupt anti-cancer treatment adherence (Berger et al., 2015). Therefore, CRF emerges as one of the most prevalent complications associated with both cancer and its treatment. It significantly compromises patient quality of life and presents a potential obstacle to successful treatment and improved survival rates.

Despite extensive research worldwide, the precise pathophysiological mechanisms underlying CRF remain elusive. This lack of clarity hinders the development of specific treatment options. Currently, interventions target various factors contributing to CRF and fall into two broad categories: non-pharmacological and pharmacological. Non-pharmacological interventions encompass lifestyle modifications, including increased physical activity, psychological support, rehabilitation programs, dietary adjustments, and sleep hygiene promotion. Pharmacological interventions involve using medications such as central nervous system stimulants (e.g., methylphenidate), agents for managing anemia (e.g., erythropoietin), antidepressants, and glucocorticoids (Kreissl et al., 2016). However, a definitive treatment strategy to completely eradicate fatigue remains elusive, highlighting the potential for natural product-based interventions.

*Cistanche tubulosa,* a venerated traditional Chinese medicinal herb, has been prized for centuries due to its purportedly targeted therapeutic effects and gentle properties. Renowned as “desert ginseng”, it has been used in Traditional Chinese Medicine (TCM) to invigorate the kidneys and nourish the body (Lei et al., 2023). Within China, four *Cistanche* species and one variety exist. *Cistanche tubulosa* stands out as the dominant medicinal resource in Xinjiang province, flourishing in arid desert regions. Notably, Xinjiang production accounts for over 80% of China’s total, making it the primary source for traditional Chinese medicine in southern Xinjiang regions like Hotan and Aksu. In recent years, *C. tubulosa* has garnered renewed interest due to its potential as a dual-purpose medicinal and edible plant, driven by its remarkable biological activity (Song et al., 2016). In recent years, research has identified total phenylethanol glycosides (CPhGs), including echinacoside and ACT, as the primary bioactive constituents and quality control markers for *C. tubulosa*. It is now understood that these CPhGs exhibit a broad spectrum of biological activities, including anti-fatigue (Kan et al., 2021), liver protection (Pei et al., 2018), kidney protection (Ding et al., 2023), anti-inflammatory (Peng et al., 2020), antioxidant (Ding et al., 2023), anti-osteoporosis (Lin et al., 2023), and anti-tumor (Wen et al., 2021) properties.

Our previous work demonstrated that ACT could promote mitophagy by suppressing PHD2, thereby upregulating the HIF-1α/BNIP3 signaling pathway and eliminating dysfunctional mitochondria, ultimately ameliorating PTX-induced CRF (Zhang et al., 2022). However, the ability of ACT to alleviate CRF and the underlying metabolic mechanisms remain to be elucidated. This study addresses this knowledge gap. We established a CRF model in mice bearing C26 colon cancer cells to evaluate the anti-CRF effects of ACT. Untargeted metabolomic profiling of mouse plasma samples was performed using liquid chromatography-mass spectrometry (LC-MS). Principal component analysis (PCA) and partial least squares-discriminant analysis (PLSDA) were employed to analyze the metabolic alterations and elucidate the potential metabolic mechanisms of ACT’s action. Ultimately, this research aims to contribute to developing novel therapeutic strategies for the benefit of cancer patients.

## 2 Materials and methods

### 2.1 Reagents

C26 colon cancer cells (CL-0071) were obtained from Procell Life Science & Technology Co., Ltd. (Wuhan, China). Acteoside (ACT; HPLC > 98.5%) was purchased from Yongjian Pharmaceutical Co., Ltd. (Taizhou, China). Paclitaxel (PTX) injection was procured from Yangtze River Pharmaceutical Group (Taizhou, China). Human CYP3A4 Protein (His Tag) and Human BCL2/Bcl-2 Protein (His Tag) were obtained from Sino Biological.

### 2.2 Animal models and drug treatment

Male BALB/c mice (16–20 g) were obtained from the Xinjiang Medical University Animal Experiment Center (certificate number SCXK [Xin] 2018-0001; SPF license number SYXK [Xin] 2018-0003). Upon arrival, the mice were housed in a specific pathogen-free (SPF) facility under controlled conditions (22°C–26°C, 40%–60% humidity, 12 h light/dark cycle) for a 7-day acclimation period with *ad libitum* access to food and water.

**FIGURE 1:**
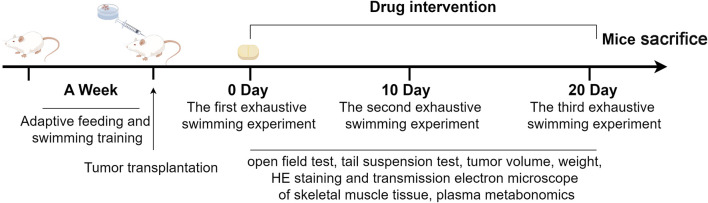
Timeline of animal experiments (by figdraw).

Male BALB/c mice were acclimated for 1 week before experimentation. During this acclimation period, the mice underwent a daily swimming adaptation training session in a high (30 cm) and diameter (25 cm) swimming box filled with water maintained at 25°C ± 1°C. Following the adaptation period, mice were randomly allocated into groups based on their swimming time (n = 10 per group). The normal control group (NC) received no C26 cell inoculation. The remaining mice were designated as the C26 group. On the day preceding inoculation, the dorsal fur of all mice designated for C26 cell injection was shaved (1 cm × 1 cm area). Log-phase C26 colon cancer cells were trypsinized and resuspended in PBS at a concentration of 1 × 10^6^ cells/mL. Each mouse received a subcutaneous abdominal injection of 0.1 mL cell suspension. Seven days post-inoculation, upon reaching a tumor volume range of 50 mm^3^–100 mm^3^, mice were again randomly divided into four groups (n = 40 per group) based on tumor volume: tumor control (TC), PTX, ACT (100 mg/kg), and PTX + ACT (100 mg/kg) groups. The ACT groups received daily intragastric administration of 100 mg/kg ACT, while the PTX groups received 10 mg/kg PTX via intraperitoneal injection every other day. Both NC and TC groups received corresponding volumes of normal saline via the same administration routes as their respective treatment groups. Treatment duration was 21 days following group allocation (7 days post C26 cell inoculation). A detailed illustration of the experimental design and procedures is provided in Figure 1.

### 2.3 Ethology assessment

A battery of behavioral tests was employed to evaluate fatigue levels across the experimental groups. These tests included the exhaustive swimming test, the open field test, and the tail suspension test.

Exhaustive swimming test: Mice underwent an exhaustive swimming test before treatment initiation and on days 10 and 20 of treatment. Lead weights equivalent to 7% of the body weight were attached to the tails of the mice. Each mouse was placed individually into a swimming box (height 30 cm, diameter 25 cm) filled with water maintained at 25°C ± 1°C. The time to exhaustion was recorded. Exhaustion was defined as the point at which the tip of the mouse’s nose submerged underwater for at least 10 s.

Open-field testing was conducted using a computerized behavioral analysis system (e.g., Chengdu Tai League TM-visual behavior experiment system). Mice were individually placed in the center of the open-field arena and allowed to freely explore for 5 min. The total distance traveled and time spent immobile were automatically recorded by the tracking software. The arena was thoroughly cleaned between trials to eliminate residual cues.

Tail suspension test: All mice in the open-field experiment were treated according to the original grouping and administration plan. 30 min after the last administration, the mouse tail (from the tail tip 2 cm) was glued to the TS-200 tail suspension tester, the mouse head was placed 5 cm from the bottom, and the immobility time was recorded for 5 min.

### 2.4 Evaluation of anti-tumor effect

Following the intervention, the longest diameter (L) and the vertical width diameter (W) of the tumor were measured with Vernier calipers every 3 days. The tumor volume (mm³) was then calculated using the formula: 0.52 × L × W^2^. One hour after the final administration, the tumor was excised and weighed. The following formula (Equation 1) was used to calculate the percentage tumor inhibition rate:

$$\text{Tumor inhibition rate}=\left(\text{average tumor weight of control group}-\text{average tumor weight of intervention group}\right)/\text{average tumor weight of control group}\times 100\%$$ (1)

### 2.5 Histological staining and transmission electron microscope of skeletal muscle

Histological staining: Skeletal muscle tissues were fixed in 10% neutral buffered formalin (NBF) after the experiment for subsequent histological analysis using light microscopy. Following fixation, 3–5 mm thick sections were deparaffinized and processed routinely for hematoxylin and eosin (H&E) staining. Images were captured using a fluorescence microscope (DMi8, Leica, Germany) and analyzed with ImageJ software.

Transmission electron microscope of skeletal muscle: Skeletal muscle tissues were post-fixed in 1% osmium tetroxide for 3 h. Following dehydration through a graded ethanol and acetone series, the samples were embedded in Spurr resin. Ultrathin sections (70 nm) were then obtained and stained with uranyl acetate and lead citrate. Finally, the sections were observed under a transmission electron microscope (TEM; JEM-1230HC, JEOL, Japan).

### 2.6 Metabolomics analysis

#### 2.6.1 Plasma metabolite extraction

24 h after the final administration, blood was extracted from the mice’s eyeballs and collected in an anticoagulant tube. The blood was then incubated at room temperature for 40 min, followed by centrifugation at 3,3000 rpm for 15 min. The upper plasma layer was separated and stored at −80°C. Prior to experimentation, plasma samples were retrieved from the −80°C freezer and thawed at 4°C. After thawing, 100 μL aliquots were transferred to 1.5 mL Eppendorf tubes, followed by the addition and thorough mixing of 500 μL acetonitrile. The supernatant was collected using a pipette, and the remaining pellet was resuspended in 100 μL of acetonitrile. This suspension was then incubated at −20°C for 2 h and centrifuged at 20,000 g for 10 min. Finally, 10 μL aliquots from each sample were combined with diluent to create quality control (QC) samples. All samples, including the QC samples, were stored at −80°C until analysis by mass spectrometry.

#### 2.6.2 LC-MS metabolomics data acquisition

The metabolomic analysis was performed on a Q Exactive mass spectrometer (Thermo Fisher Scientific, USA) at LC-Bio Technologies (Hangzhou) Co., Ltd. To minimize batch effects, samples from all groups were interspersed throughout the LC-MS run order. Chromatographic separations were achieved using a Thermo Scientific UltiMate 3000 HPLC system equipped with an ACQUITY UPLC T3 column (100 mm × 2.1 mm, 1.8 μm particle size; Waters, UK) maintained at 50°C for reversed-phase chromatography. The mobile phase consisted of 0.1% formic acid in water (phase A) and 0.1% formic acid in acetonitrile (phase B). A gradient elution program was employed: 0–0.8 min, 2% B; 0.8–2.8 min, 2%–70% B; 2.8–5.6 min, 70%–90% B; 5.6–8 min, 90%–100% B; 8–8.1 min, 100%–2% B; 8.1–10 min, 2% B. The injection volume was 4 μL per sample. The instrument wash cycle included one or two washes and 3–4 QC samples. Subsequently, a QC sample was injected after every 10 samples, and the run concluded with two final QC injections. High-resolution tandem mass spectrometry using the Q-Exactive instrument facilitated the acquisition of first and second-order mass spectra for eluting metabolites in both positive and negative ionization modes. Precursor ion scans (m/z 70–1050) were acquired at a resolution of 70,000 with an AGC target of 3 × 10^6^ and a maximum injection time of 100 ms. Data-dependent acquisition (DDA) mode was employed with a “top 3” configuration. Fragment spectra were collected at a resolution of 17,500 with an AGC target of 1 × 10^5^ and a maximum injection time of 50 ms. Stepped normalized collision energy (nce) settings for fragmentation were 20, 40, and 60 eV. The electrospray ionization (ESI) source parameters were set as follows: spray voltage (|KV|) 4000 (positive and negative modes), sheath gas flow rate 35, auxiliary gas flow rate 10, and capillary temperature 320°C.

#### 2.6.3 Metabolomics data processing and analysis

Mass spectrometry raw data files (.raw) acquired from the experiment were imported into Compound Discoverer 3.1.0 (Thermo Fisher Scientific, USA) for data pre-processing. This pre-processing included peak extraction, retention time correction within and between sample groups, adduct ion merging, gap filling, background peak labeling, and metabolite identification. Each metabolite ion was identified by its retention time (RT) and m/z value. Peak intensities were recorded for all identified metabolites. Finally, information such as feature molecular weight, retention time, peak area, and identification results were exported for further analysis. Online databases, including KEGG and HMDB, were utilized for metabolite annotation. This annotation process involved matching the exact molecular mass data, names, and formulas of the identified metabolites with database entries. Metabolites were considered tentatively identified if the mass difference between the observed and database values was less than 10 ppm.

Following peak data acquisition, further preprocessing was performed using metaX software. Features detected in less than 50% of QC samples or 80% of biological samples were removed to ensure data quality. Missing values in the remaining peaks were imputed using the k-nearest neighbor (kNN) algorithm to improve data completeness. Principal component analysis (PCA) was then performed on the preprocessed data to identify outliers and evaluate potential batch effects. Probabilistic quotient normalization (PQN) was subsequently employed to normalize the data, resulting in normalized ion intensity data for each sample. To minimize signal intensity drift over time, QC-based robust locally estimated scatterplot smoothing (LOESS) signal correction was applied to the QC data, considering the injection order. Finally, the coefficient of variation (CV) was calculated for each metabolic feature across all QC samples. Features with a CV exceeding 30% were removed from further analysis.

Student’s t-tests were used to identify metabolites with significantly different concentrations between the two phenotypes. P-values were adjusted for multiple comparisons using the Benjamini–Hochberg procedure. To further discriminate between groups and identify important features, supervised partial least squares-discriminant analysis (PLS-DA) was performed via MetaX software. Metabolites with a Variable Importance in Projection (VIP) score greater than 1.0 and a fold-change greater than or equal to 2 or less than or equal to 0.5 (*p*-value ≤0.05) were subjected to KEGG enrichment analysis.

#### 2.6.4 Pathway enrichment and protein network analysis

To elucidate the origin and function of differentially abundant metabolites, metabolic pathway enrichment analysis was performed using the online tool MetaboAnalyst 5.0. Pathways with a p-value threshold of less than 0.05 were considered statistically significant and enriched. To identify key proteins potentially linking the effects of ACT to these altered metabolites, a protein-protein interaction (PPI) network was constructed based on proteins associated with the differential metabolites. Briefly, the HMDB 5.0 protein data (downloaded in XML format on 24 September 2021) was retrieved locally. Subsequently, a custom R script was employed to extract metabolite-related proteins from the STRING database.

### 2.7 Surface plasmon resonance (SPR) assay

SPR analysis was conducted with the Open SPR instrument (Nic-oyalife, Canada). The NTA sensor chip was first installed on theOpenSPR instrument in accordance with the standard operating procedure.

#### 2.7.1 Ligand immobilization

The activator is prepared by mixing 400 mM EDC and 100 mM NHS immediately prior to injectionThe chip is activated for 240 s with the mixture at a flow rate of 20 μL/min. Dilute BCL2 to 50 μg/mL inimmobilization buffer, then injected to sample channel at a flow rate of 20 μL/min. The chip isdeactivated by 1 M Ethanolamine hydrochloride at a flow rate of 20 uL/min for 240s.The activator is prepared by mixing 400 mM EDC and 100 mM NHS immediately prior to injection. The chip is activated for 240 s with the mixture at a flow rate of 20 pL/min. Dilute CYP3A4 to 50 µg/mlin immobilization buffer, then injected to sample channel at a flow rate of 20 μL/min. The chip isdeactivated by 1 M Ethanolamine hydrochloride at a flow rate of 20 μL/min for 240 s.

#### 2.7.2 Running analyte by multi-cycle method

Dilute Acteoside with the same analyte buffer buffer to 7 concentrations (500, 250, 125, 62.5, 31.2515.625 and 0 ylM). Acteoside is injected to sample channel at a flow rate of 20 uL/min for an associationphase of 240 s, followed by 360 s dissociation. The association and dissociation process are all handling inthe analyte buffer. Repeat 7 cycles of analyte according to analyte concentrations in ascending orderA fter each cycle of interaction analysis, the sensor chip surface should be regenerated completely with 10 mM Glycine-HCl as injection buffer at a flow rate of 1 50 uL/min for 10 s to remove the analyte, then nextconcentration cycle ofthe analyte Acteoside need to repeat injection and regeneration steps.

### 2.8 Statistical analysis

Statistical analysis of ethology assessment and anti-tumor effect evaluation was performed using one-way ANOVA followed by an LSD post-hoc test in SPSS software (version 16, SPSS Inc., Chicago, IL). Data were presented as mean ± standard deviation (SD). Statistical significance was set at *p* < 0.05. Column plots were generated using GraphPad Prism (version 7.00, GraphPad Software Inc., La Jolla, CA).

A heatmap, with color intensity reflecting the abundance, was used to represent the relative abundance of metabolites detected in each sample. The heatmap was generated using the pheatmap function within the pheatmap R package (version 1.0.12).

## 3 Results

### 3.1 Effect of ACT on the general condition of tumor-bearing mice

Prior to the intervention, no significant differences in mental state, activity level, diet, or fur appearance were observed among the five groups. All mice exhibited liveliness, activity, smooth and well-groomed fur, bright eyes, and a keen response to food and escape stimuli. Throughout the experiment, the mice in the NC group maintained their liveliness, activity, neat and smooth fur, food sensitivity, and rapid escape response. In contrast, the four tumor-bearing groups progressively displayed signs of hair loss, inactivity, sluggish movement, and decreased food intake compared to the NC group. At the end of the experiment, the overall condition of the four tumor-bearing groups, ranked from poorest to relatively best, were: TC + PTX, TC, TC + PTX + ACT, and TC + ACT.

### 3.2 Effect of ACT on exhaustive swimming time

**FIGURE 2:**
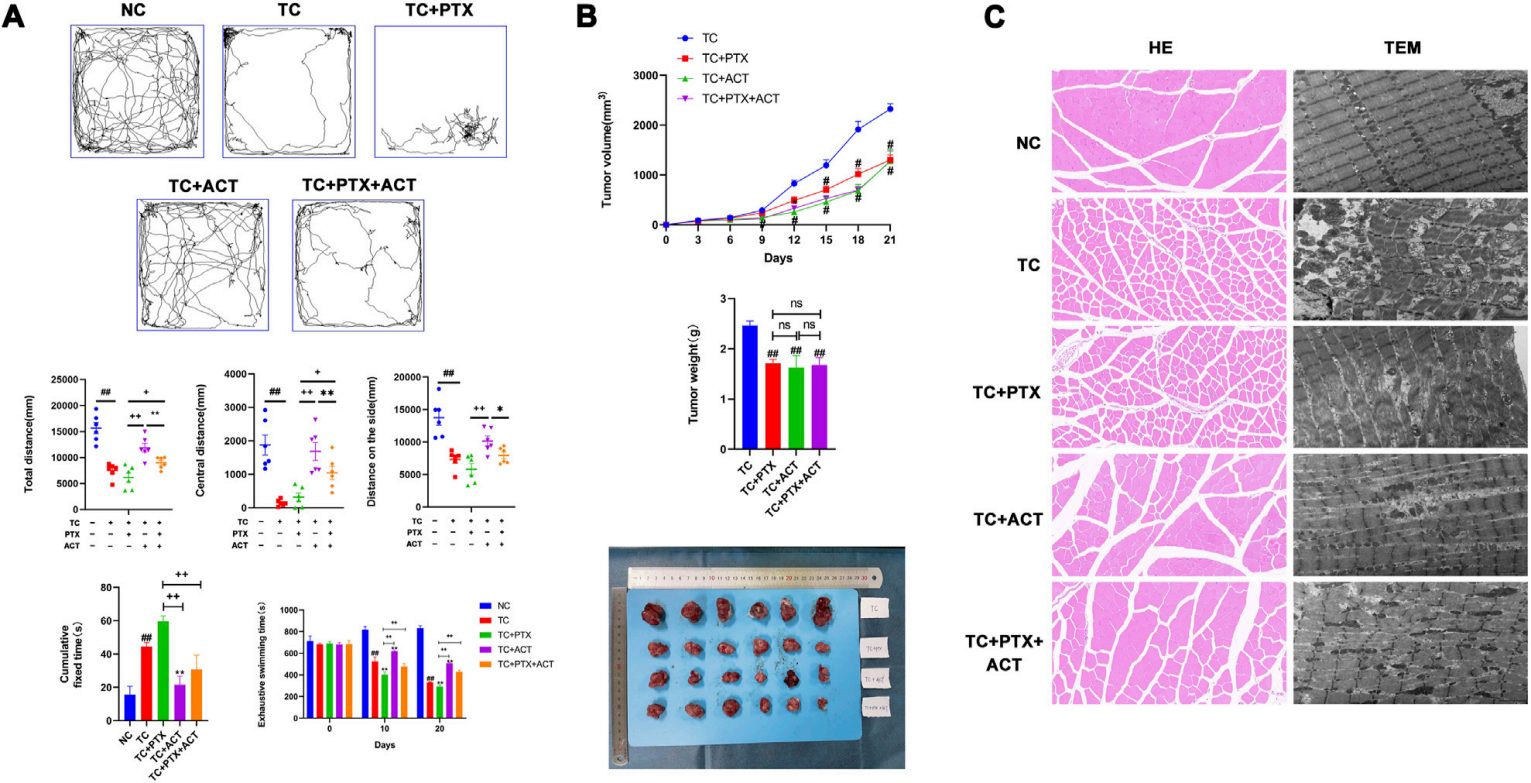
Alleviating effect of ACT on colon cancer cell-induced CRF. 100 mg/kg ACT was administered intragastrically, once daily. 10 mg/kg PTX was administered by intraperitoneal injection once every 2 days. **(A)** The results of the open field test in each group of mice, the degree of fatigue was evaluated by the exhaustive swimming time test and the results of the tail suspension test in each group of mice (Mice struggled to escape for a period of time and adopted a posture of immobility. The cumulative immobility time of the animal was recorded during the experiment); **(B)** E Effect of ACT on tumor size, volume and weight in tumor-bearing mice. **(C)** Photomicrographs of skeletal muscle sections stained with H&E (200X), representative images of the mitochondrial ultrastructure in skeletal muscle sections under transmission electron microscopy (scale bar = 2 µm). The values are presented as mean ± SD. #*p* < 0.05, ##*p* < 0.01, compared with NC group, ^+^ *p* < 0.05, ^++^ *p* < 0.01, compared with TC + PTX group, *p < 0.05, **p < 0.01, compared with TC + PTX + ACT group.

Exhaustive swimming tests were employed alongside the open field test and tail-suspension test to assess the anti-fatigue effects of ACT (Figure 2A). Before the intervention, all groups displayed similar exhaustive swimming times. However, on day 10, tumor-bearing mice in the treatment group exhibited a significantly reduced exhaustive swimming time compared to the control group. Interestingly, the NC group showed a significantly prolonged exhaustive swimming time compared to the tumor-bearing group (TC). The TC + PTX group displayed a significant decrease in exhaustive swimming time compared to the TC group, while the TC + ACT group showed a significant increase. Notably, no significant difference was observed between the TC + PTX + ACT group and the TC group. Compared to the TC + PTX group, all other groups exhibited significantly prolonged exhaustive swimming times. On day 20, tumor-bearing mice in the NC group again demonstrated a significant decrease in exhaustive swimming time compared to the NC group. Compared to the TC group, the NC group and TC + ACT group displayed significantly longer exhaustive swimming times, while the TC + PTX + ACT group showed no significant difference. Additionally, the NC group, TC + PTX + ACT group, and TC + ACT group exhibited significantly longer exhaustive swimming times than the TC + PTX group.

### 3.3 Effect of ACT on the open field test

The open field test revealed that the total movement distance, peripheral movement distance, and center movement distance measured over 5 min were all significantly lower in the TC and TC + PTX groups compared to the NC group (Figure 2A). Interestingly, the peripheral movement distance in the TC + PTX + ACT group was significantly higher than that observed in both the TC and TC + PTX groups.

### 3.4 Effect of ACT on the tail suspension experiment

The tail suspension test, as depicted in Figure 2A, demonstrated that mice in the TC and TC + PTX groups exhibited significantly longer immobility than the NC group. Notably, both the TC + PTX + ACT and TC + ACT groups displayed significantly shorter immobility times compared to the TC group (*p* < 0.05).

### 3.5 Inhibitory effect of ACT on tumor growth in tumor-bearing mice

A two-factor repeated-measures analysis of variance (ANOVA) was conducted to assess tumor volume in mice across different time points. This analysis revealed significant main effects of time (*p* < 0.01), indicating that tumor volumes differed significantly before and after intervention. Additionally, significant main effects of groups were observed (*p* < 0.05), suggesting differences in tumor volume between the mouse groups. Furthermore, the interaction between time and group was significant (*p* < 0.01), implying that the effect of intervention on tumor volume varied across the different groups (Figure 2B).

At the end of the experiment, the tumor weights of the TC + PTX, TC + ACT, and TC + PTX + ACT groups were all significantly lower compared to the TC group (*p* < 0.01). Consistent with this observation, the tumor inhibition rates were 30.49%, 33.92%, and 31.77% for the TC + PTX, TC + ACT, and TC + PTX + ACT groups, respectively.

### 3.6 Histopathology changes of the effect of ACT on skeletal muscle

Hematoxylin and eosin (H&E) staining revealed normal muscle tissue architecture in the NC group, with intact adventitia, muscle fibers, and myoglobin content (Figure 2C). In contrast, the TC and TC + PTX groups displayed several abnormalities, including bent muscle fibers, dissolved myoglobin, pyknotic or dissolved muscle nuclei, and ruptured muscle fascicles. The TC + PTX + ACT group showed a limited degree of muscle fiber disruption, with a few fibers exhibiting breakage, dissolution, and bending. Interestingly, the TC + ACT group displayed a similar injury pattern to a small extent, with the remaining muscle fibers appearing normal.

### 3.7 Transmission electron microscope changes of the effect of ACT on the skeletal muscle ultrastructure

Transmission electron microscopy (TEM) analysis of skeletal muscle in the NC group revealed a normal Z-line structure characterized by smoothness. Notably, mitochondria were evenly distributed on both sides of the Z-line, exhibiting a long, linear, or oval shape with well-defined cristae (Figure 2C). In contrast, the TC and TC + PTX groups displayed significant mitochondrial abnormalities. These included an irregular Z-line, uneven mitochondrial distribution, and altered mitochondrial morphology (shape and size variations). Additionally, mitochondrial swelling and a loss of cristae structure were observed in these groups. The TC + PTX + ACT group demonstrated partial recovery of mitochondrial morphology, with the reappearance of some missing cristae. Interestingly, the TC + ACT group also exhibited a partial restoration of mitochondrial shape, with some mitochondria regaining a slender rod or oval form, suggesting an alleviation of mitochondrial damage in this group.

### 3.8 Metabolomics data processing and multivariate analysis

**FIGURE 3:**
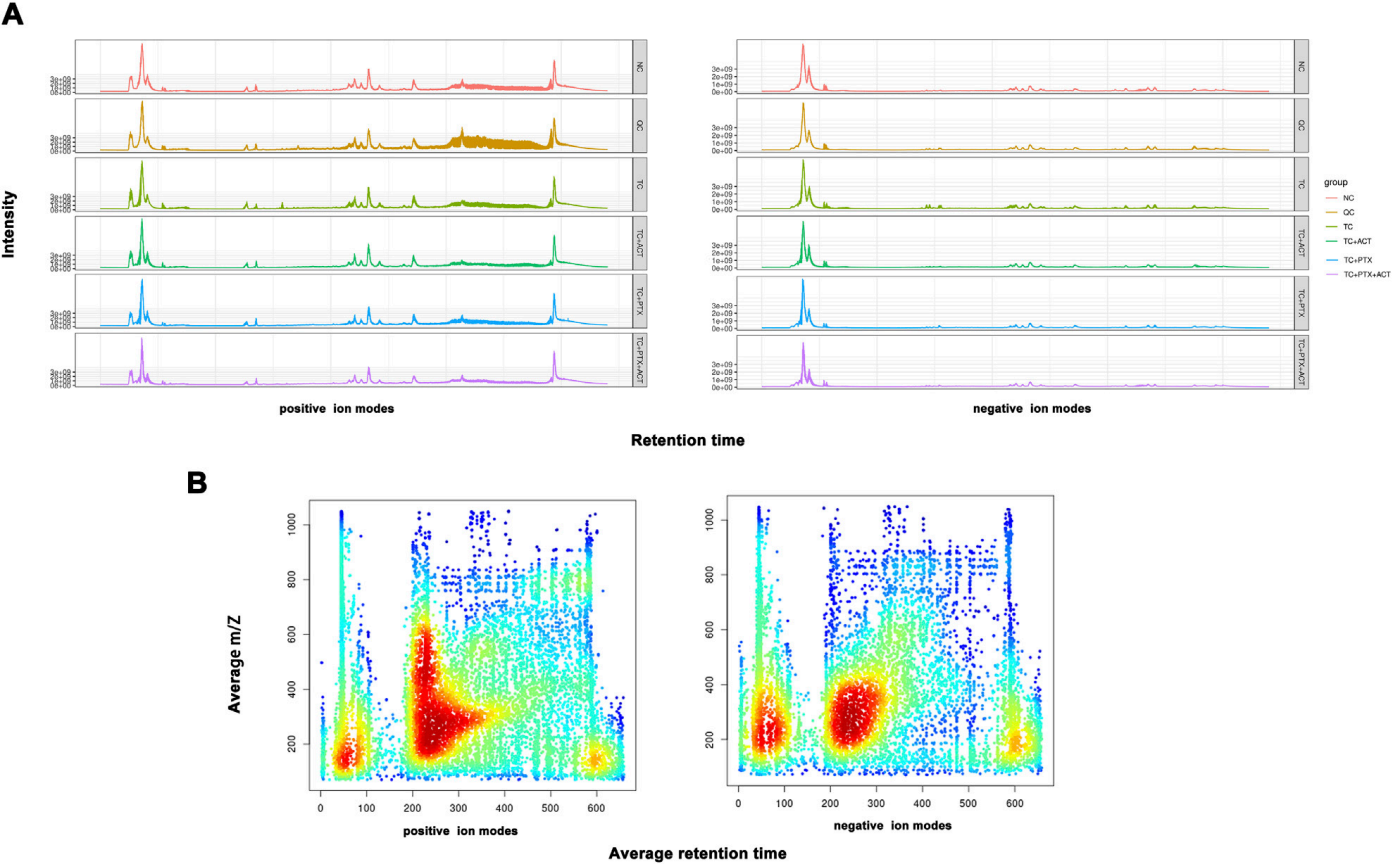
**(A)** The total icon Chromatogram of each sample is drawn based on the ion strength, which is drawn continuously with the time point as the Abscissa and the sum of all the ions in the mass spectrum as the ordinate), and the signal intensity of the quality spectrum of the sample can be controlled overall. **(B)** The distribution of total ions was analyzed in the dimensions of charge-mass ratio (M/Z) and Retention time. The material retention time was represented by Abscissa, and the mz-rt distribution was plotted by ordinate m/z. Each point in the map represents an ion, and the color indicates the concentration of ions in this area.

Building on our previous pharmacodynamic findings demonstrating the ability of ACT to alleviate CRF in C26 colon cancer cachectic mice, this study employs a non-targeted liquid chromatography-mass spectrometry (LC/MS)-based metabonomics approach. This approach aims to elucidate the effects of ACT on the serum metabolic profile of these mice and unveil its underlying mechanism in CRF prevention and treatment from a metabolic perspective. To ensure data quality, we performed quality control measures. The total ion chromatograms (TICs) of all quality control (QC) samples were overlaid to assess the stability of the mass spectrometer during ion collection, as shown in Figures 3A, B. A heatmap analysis with hierarchical clustering of all the groups is displayed in Supplementary Figure S1. The high degree of spectral overlap, minimal fluctuations in retention time, and consistent peak response intensity across QC samples indicate excellent instrument performance and stable signal throughout sample detection and analysis.

**FIGURE 4:**
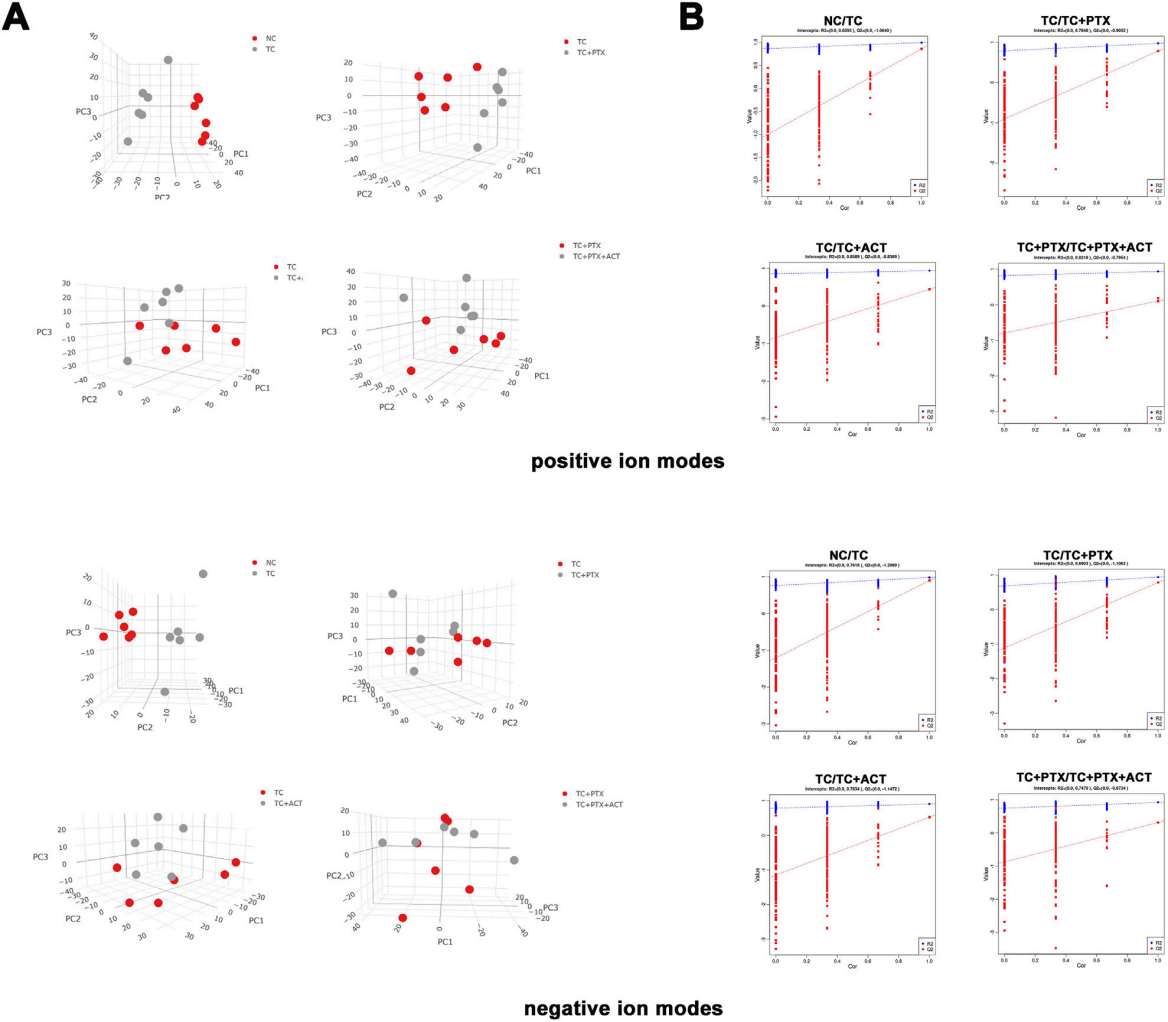
**(A)** PCA score scatter plots (3D) showing clustering of plasma metabolites detected in ESI- mode (POS) and ESI + mode (NEG). **(B)** PLS-DA score scatter plots showing clustering of plasma metabolites detected in ESI- mode (POS) and ESI + mode (NEG). NC vs. TC (POS, R2Y = 0.8595, Q2 = −1.004; NEG, R2Y = 0.7618, Q2 = −1.2069), TC vs. TC + PTX (POS, R2Y = 0.7848, Q2 = −0.9052; NEG, R2Y = 0.6903, Q2 = −1.1063), TC vs. TC + ACT (POS, R2Y = 0.8589, Q2 = −0.8369; NEG, R2Y = 0.7834, Q2 = −1.1472), TC + PTX vs. TC + PTX + ACT (POS, R2Y = 0.8218, Q2 = −0.7954; NEG, R2Y = 0.0.747, Q2 = −0.8734).

Metabonomic studies often utilize multivariate statistical analysis techniques such as PCA and partial least squares-discriminant analysis (PLS-DA). In this investigation, PCA and PLS-DA were employed to analyze the metabolic profile differences in mice with CRF following ACT treatment to identify differential metabolites. The 3D PCA score plot is presented in Figure 4A. The 2D version of the PCA plot is displayed in Supplementary Figure S2. Both positive and negative ion mode analyses revealed significant separation in the plasma metabolic profiles between the NC and TC groups, as well as between the TC and TC + PTX groups, indicating distinct metabolic profiles in CRF mice compared to the NC group. Additionally, the plasma metabolic profile of mice treated with the chemotherapeutic drug PTX significantly differed from that of the tumor cell-induced late-stage cachexia model mice. Furthermore, significant differences were observed between the TC and TC + ACT groups, as well as between the TC + PTX and TC + PTX + ACT groups. Taken together, these findings suggest that ACT treatment for 21 days exerted a significant influence on the serum profile of CRF mice, suggesting that ACT may alleviate CRF progression by regulating metabolic disturbances associated with advanced cachexia.

To identify potential metabolic markers associated with ACT treatment, PLS-DA was employed to further analyze the differential metabolites between the TC and TC + ACT groups, as well as between the TC + PTX and TC + PTX + ACT groups (Figure 4B), and the VIP metabolites PLS-DA plots for each group are displayed in Supplementary Figure S3. The PLS-DA model exhibited significant separation between the NC and TC groups, and between the TC and TC + PTX groups. Additionally, the model demonstrated good fitting and high predictive ability. These findings collectively suggest distinct metabolic profiles in tumor cell-induced CRF mice compared to the NC group and further support the presence of metabolic differences between mice treated with PTX and those in the CRF model. Furthermore, the PLS-DA results revealed significant differences between the TC + ACT and TC groups, as well as between the TC + PTX + ACT and TC + PTX groups, indicating that ACT treatment for 21 days significantly modulated the serum metabolic profile of mice in both the CRF model group and the chemotherapy drug PTX group.

### 3.9 Screening and identifying differential metabolites

To identify the key features primarily responsible for group separation, a three-tiered screening process was implemented for differential variable selection. First, univariate analysis using Student's t-test was employed to identify features with statistically significant differences (*p*-value < 0.05) between the two groups being compared. Second, VIP scores were used to assess the contribution of each variable to the model’s ability to differentiate the groups. Variables with VIP scores greater than 1 were considered potentially important. Finally, a fold-change filter was applied, retaining only features that exhibited a fold-change greater than or equal to 2 or less than or equal to 1/2 in abundance between the compared groups. This selection process yielded a distinct number of differential variables in both positive (POS) and negative (NEG) ionization modes. Notably, the number of differential variables was consistently higher in the positive ionization mode (e.g., 264 vs. 167 for NC-TC comparison) compared to the negative ionization mode. This observation might be attributed to the preferential detection of endogenous plasma metabolites in positive ionization mode.

**FIGURE 5:**
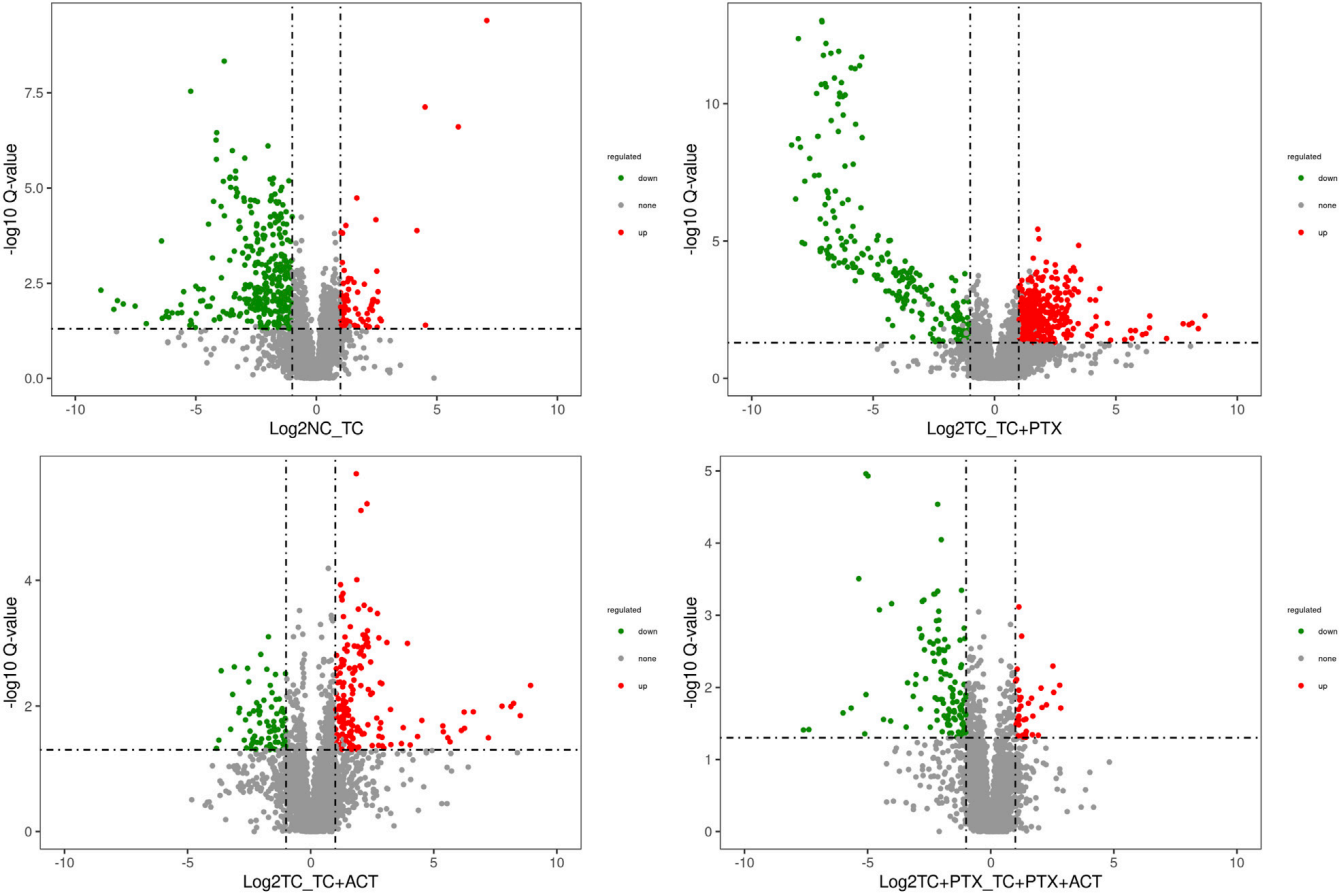
Volcano plot depicting the distribution of significantly altered metabolites. The log2 (NC_TC), log2 (TC_TC + ACT), log2 (TC_TC + PTX), and log2 (TC + PTX_TC + PTX + ACT) represent the mean level for each metabolite, respectively. Each dot represents one metabolite. Blue and red dots represent significantly altered metabolites, and black dots represent non-significantly altered metabolites.

**FIGURE 6:**
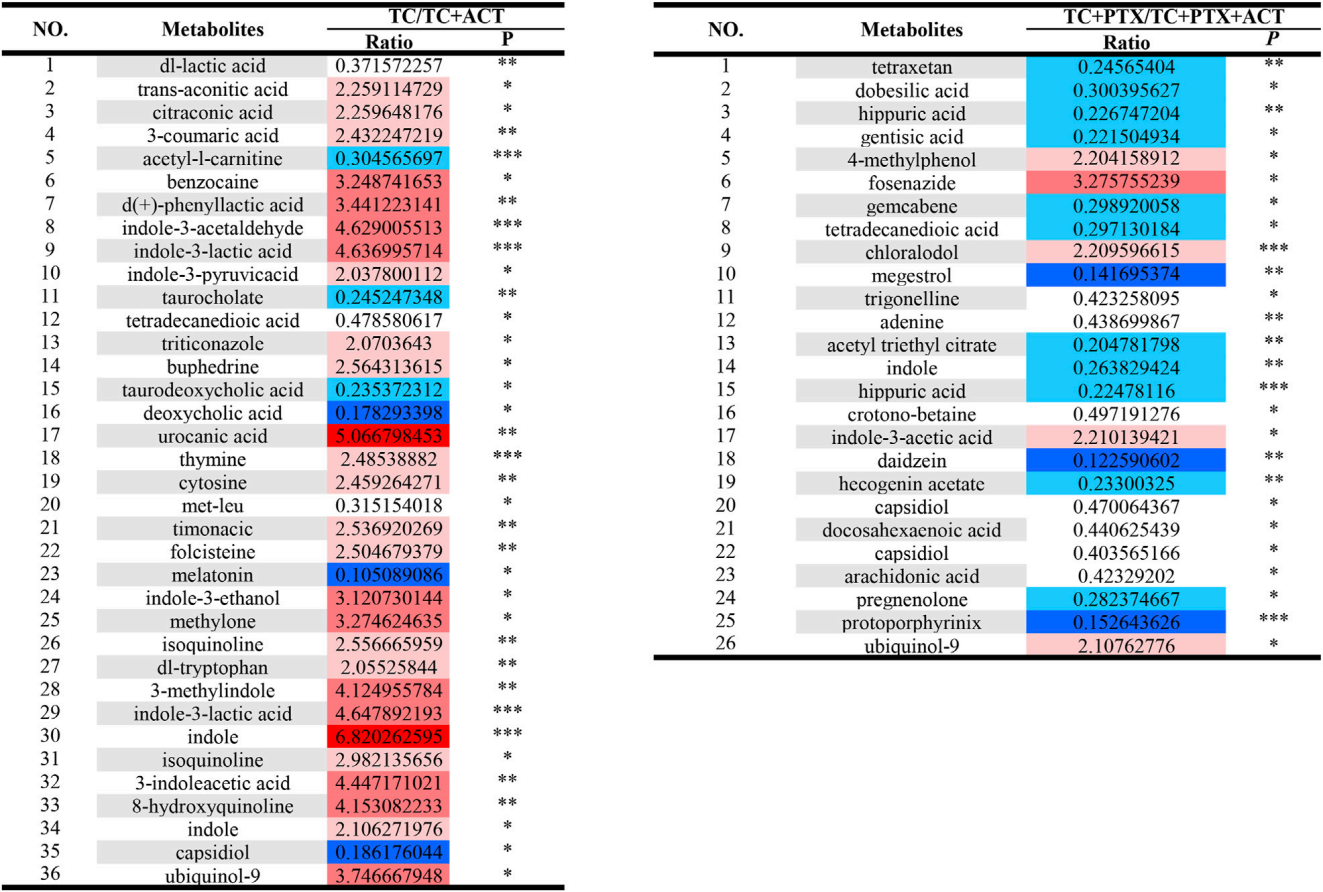
Univariate analysis Color Table: the color coding is performed according to the change in magnification (FC). The darker the color, the higher the significance; the darker the red, the higher the positive magnification of the difference between the two groups; and the darker the blue, the higher the reverse magnification of the difference between the two groups. The *t*-test or non-parametric Mann-Whitney test was performed, and the *p* values were corrected by the Benjamini–Hochberg method, *p < 0.05, **p < 0.01, ***p < 0.001.

Figures 5, 6 depict the profile and abundance of differentially expressed metabolites. Figure 5 illustrates that 15 potential biomarkers exhibited increased levels in the TC group, except for dl-lactic acid and fumaric acid, which showed a decrease. Notably, administration of ACT reversed these trends, restoring dl-lactic acid, trans-aconitine, citric acid, 3-coumaric acid, dl-tryptophan, and indole isoquinoline to normal levels.

Blood analysis of tumor-bearing mice (TC group) revealed significantly elevated levels of several metabolites compared to the NC group. These metabolites included dl-malic acid, dl-lactic acid, d-(−)-erythrose, L-threose, fumaric acid, and acetyl-L-carnitine. The increase in acetyl-L-carnitine, an ester of L-carnitine primarily found in muscle tissues, is noteworthy. Acetyl-L-carnitine is known to promote fatty acid oxidation, enhance cellular protection, and inhibit lactic acid accumulation. The observed muscle atrophy in cachectic tumor-bearing mice, characterized by fatigue and weakness, suggests a potential increase in skeletal muscle fat catabolism, which may be linked to elevated lactic acid levels. Previous studies have established a link between overweight/obesity and the development of colorectal cancer. Additionally, obesity has been shown to influence colorectal cancer growth and metastasis, further supporting the notion of disrupted fat metabolism in colorectal cancer patients. Interestingly, the levels of metabolites associated with fatty acid metabolism (lauric acid, isovalerate, and nervonic acid) were decreased in the colorectal cancer mice. This finding suggests a potential upregulation of anaerobic respiration in these tumors, possibly contributing to increased lactic acid. Consequently, high lactic acid levels may represent one of the mechanisms underlying colorectal cancer development in this mouse model.

Compared to the TC group, the TC + PTX group exhibited increased levels of several metabolites, including cis-aconitonic acid, itaconic acid, quinolinic acid, trans-aconitonic acid, citric acid, and glycine. Conversely, the levels of octanoyl amino acid, cholic acid, β-cholic acid, deoxycholic acid, histamine, β-alanine, and cortisol were downregulated in the TC + PTX group. PTX is a novel microtubule-targeting agent that exerts its antitumor activity by promoting tubulin polymerization, inhibiting depolymerization, and stabilizing microtubules, ultimately leading to cell cycle arrest and mitotic inhibition. PTX demonstrates significant clinical efficacy in treating various tumors. However, it is well-known that PTX treatment can cause neurotoxicity and muscle/joint pain, which are considered significant contributors to chemotherapy-induced fatigue.

### 3.10 Metabolic pathway analysis

**FIGURE 7:**
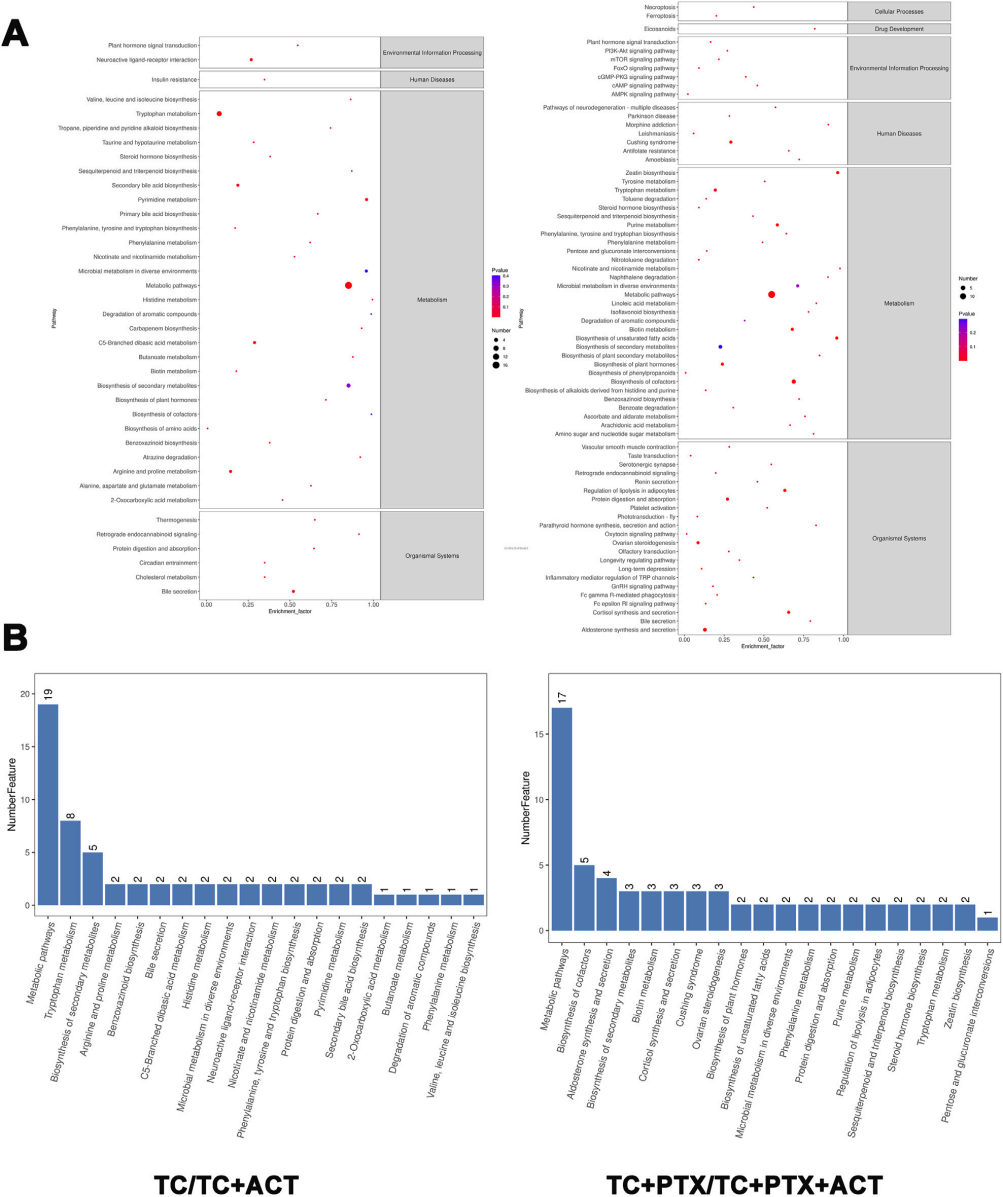
KEGG enrichment circle diagram and bubble diagram. **(A)** Information on the environmental processing, human diseases, cellular processes, and organismal systems of the KEGG enrichment. **(B)** The metabolic pathway of the top 20 enrichment. The ordinate is the pathway, and the abscissa is the enrichment factor. The size indicates the quantity. The redder the color, the smaller the Q value, the higher the significance.

To elucidate the metabolic pathways associated with the identified differential metabolites, enrichment analysis was performed using the MetaboAnalyst tool (Figure 7). This analysis revealed that the altered metabolites participate in a diverse range of pathways, including biosynthesis of branched-chain amino acids (valine, leucine, and isoleucine), ubiquinone and other terpenoids, and phenylenediones. Additionally, pathways involved in tyrosine and tryptophan metabolism, biosynthesis of alkaloids, toluene degradation, taurine and hypotaurine metabolism, steroid hormone biosynthesis, secondary bile acid biosynthesis, pyrimidine metabolism, thiamine metabolism, purine metabolism, propionic acid metabolism, porphyrin and chlorophyll metabolism, and ketone body synthesis and degradation were also enriched. This comprehensive analysis suggests that ACT treatment may exert its effects by modulating a broad network of metabolic pathways.

### 3.11 Analysis of interactions between proteins related to metabolites

**FIGURE 8:**
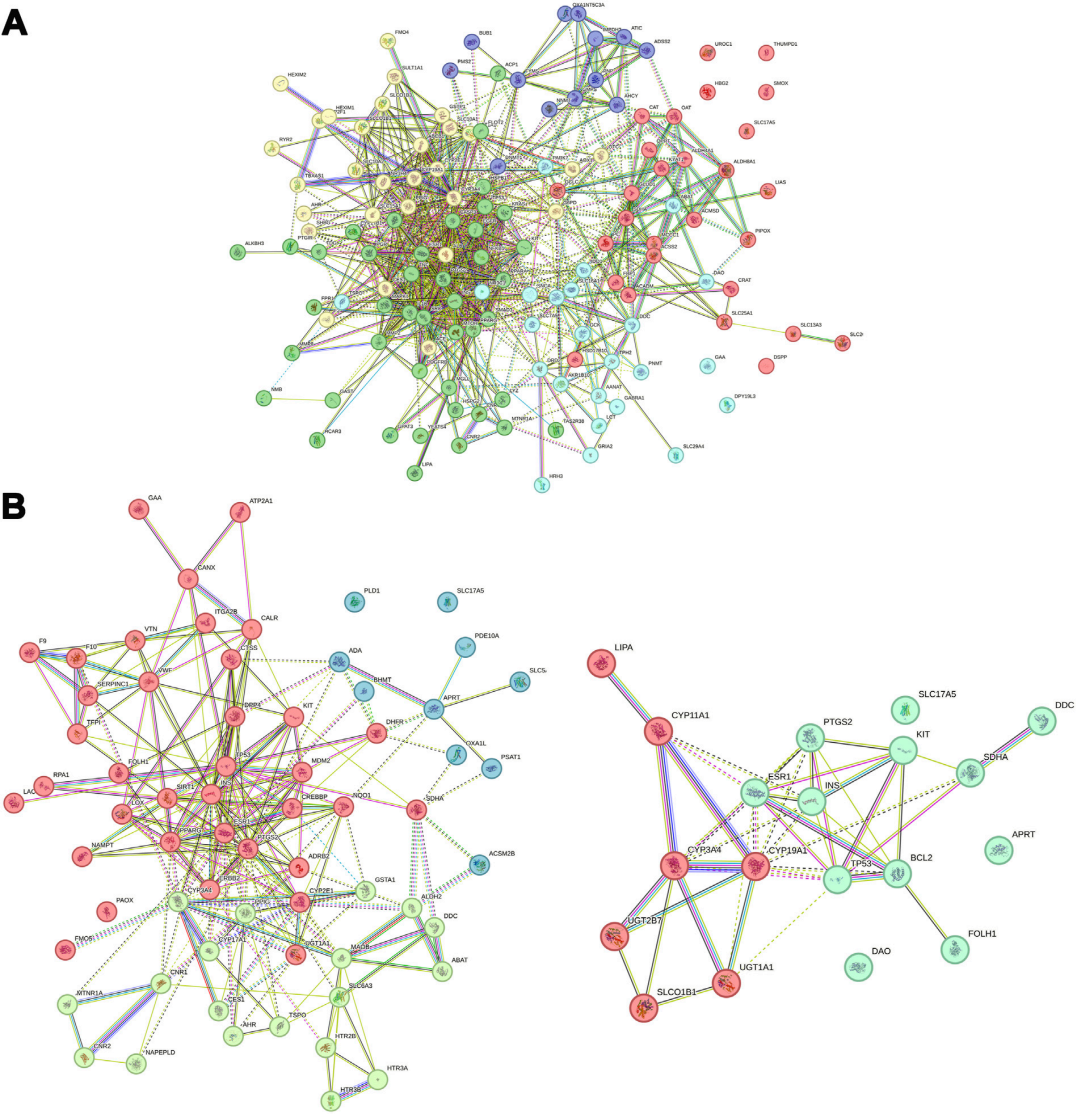
PPI network analysis of differential metabolites-related proteins. **(A)** TC vs. TC + ACT, **(B)** TC + PTX vs. TC + PTX + ACT.

Building upon the previously described approach, this analysis aimed to identify potential interactions between metabolites and their associated proteins, as well as relationships between the proteins themselves. A total of 137 and 84 proteins were found to be associated with differential metabolites between the TC and TC + ACT groups and between the TC + PTX and TC + PTX + ACT groups, respectively. A PPI network was constructed using the visNetwork software, and the results are presented visually in Figures 8A, B. The identified hub proteins in the TC vs. TC + ACT comparison included CYP3A4, CYP19A1, CYP2E1, TNF, MAPK1, IL-1, SMAD3, MMP2, FMO4, SLC7A5, SLC16A1, SLC29A4, PDGFR, HSPG2, and RYR2. Notably, the hub proteins in the TC + PTX vs. TC + PTX + ACT comparison were BCL-2, CYP11A1, CYP3A4, CYP19A1, SLC17A5, SLC6A3, PTGS2, DPP4, PLD1, ATP2A1, LOX, PPARG, and FMO5.

**FIGURE 9:**
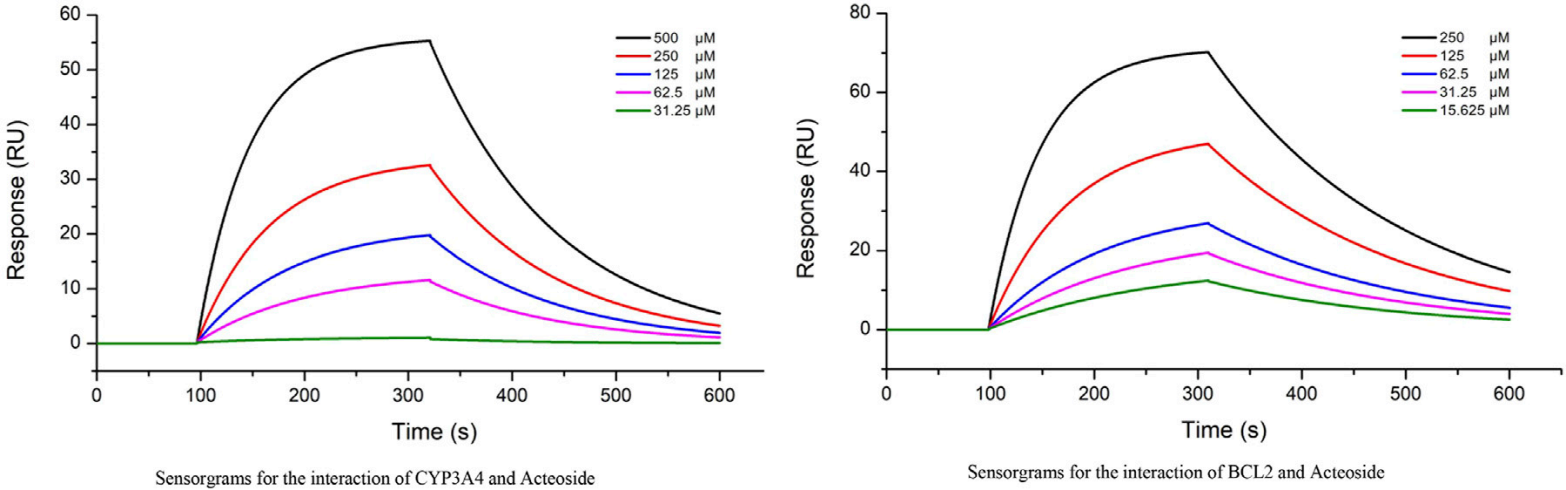
Interactions between the ACT and CYP3A4, BCL-2 were determined by SPR assay, The KD of the CYP3A4 and BCL-2 protein with a series of concentrations of ACT was calculated by SPR.

The interactions between ACT and CYP3A4,BCL-2 (two groups of top ranked genes) were further evaluated by real-time biomolecular interaction analysis and SPR. The kinetics of the binding reaction were determined by injecting different concentrations of the compounds over a recombinant human CYP3A4 and BCL-2 immobilized on the chip surface. The data were fitted to a monovalent binding model by non-linear regression. The equilibrium dissociation constants (KD) for ACT and CYP3A4 were 3.41E-4, respectively, with association rates of 4.09e2 and dissociation rates of 8.24E-3. The equilibrium dissociation constants (KD) for ACT and BCL-2 were 8.81E-5, respectively, with association rates of 4.09e2 and dissociation rates of 5.41E-3 (Figure 9).

## 4 Discussion

With the development of society, the incidence of tumors is steadily increasing. However, advancements in diagnosis and treatment technologies, coupled with heightened public awareness of self-care, have improved tumor prevention and treatment strategies, resulting in a relative increase in patient survival rates. As an increasing number of people will experience tumors at some point in their lives, ensuring their quality of life becomes even more critical. CRF, one of the most common complications associated with both tumors and their treatment, significantly impacts patient quality of life and creates a burden on tumor treatment and survival. Despite extensive research by scholars and clinicians worldwide, the exact pathophysiological mechanisms of CRF remain unclear, and there is a lack of widely recognized, effective interventions in modern medicine (Wang and Woodruff, 2015). TCM, however, offers unique advantages and demonstrable efficacy in tumor treatment. The clinical application of TCM can reduce the side effects of chemotherapy, enhance the efficacy of anti-tumor therapies, and improve patients’ immunity (Najafi et al., 2021). Based on these advantages, this study investigates the pharmacological effects of a specific ACT compound derived from *C. tubulosa* on reducing fatigue in colon cancer mice, utilizing a metabonomic approach. This research provides valuable experimental data for the potential clinical application of ACT intervention, which holds great significance for improving the quality of life of tumor patients.

A disease model is a valuable tool for studying the pathogenesis of a disease. Three main approaches are commonly employed to establish CRF models in mice. One common method involves inducing CRF in normal mice using chemotherapeutic drugs. This approach replicates the fatigue experienced by clinical tumor patients undergoing chemotherapy due to drug toxicity and side effects, including anemia. Propranolol can further induce fatigue like that observed in patients (Norden et al., 2015a; Norden et al., 2015b). The first model, induced by chemotherapeutic drugs, primarily relies on behavioral tests to assess fatigue in mice undergoing tumor treatment. This approach not only helps gauge the fatigue state but also facilitates the identification of relevant target tissues. This is particularly valuable in elucidating the specific tissue damage caused by different chemotherapeutic drugs and their contribution to fatigue. The second model utilizes peripheral radiation to induce CRF. Unlike the first model, radiation-induced CRF excludes factors like weight loss, anemia, and emotional distress, mimicking the fatigue experienced by patients with non-metastatic cancers following localized radiotherapy. This model offers insights into the biological mechanisms underlying fatigue after radiotherapy. However, due to stringent regulations on radioactive materials and limited access to specialized equipment in China, research using this model is comparatively scarce (Renner et al., 2016). The third model employs tumor-bearing mice in a state of late-stage cachexia. This is achieved by inoculating the mice with exogenous tumor cells. Since fatigue in mice is primarily linked to the decline in bodily functions observed in the late stages of cancer, this model effectively simulates the fatigue mechanism associated with cachexia in patients with advanced tumors. Additionally, the model utilizes behavioral tests to monitor changes in mouse activity. By subcutaneously injecting exogenous tumor cells, researchers can mimic clinical tumors at various stages. This allows for dynamic observation of the fatigue state in mice throughout the progression of the tumor, from formation to natural death. This closely aligns with the clinical experience of patients with advanced malignant tumors who are ineligible for radiotherapy and chemotherapy. Therefore, this model offers significant reference value for investigating severe fatigue in advanced tumor-bearing mice (Dougherty et al., 2017). Based on our previous findings demonstrating that a specific ACT compound could significantly improve CRF induced by the chemotherapeutic drug PTX, this study utilized a model established by inoculating mice with colon cancer cells to assess fatigue through open field, tail suspension, and exhaustive swimming tests.

To select an appropriate evaluation method for the CRF animal model, we conducted a comprehensive analysis of the main clinical causes of CRF in this context. These causes include 1) Direct tumor influence: Tumor cells produce cytokines like IL-1, IL-2, IL-6, and TNF-α, promoting tumor growth, disrupting normal cellular metabolism, and compromising bodily functions (Bower and Lamkin, 2013). 2) Treatment factors: The increasing use of combination therapy, dose-intensive regimens, and dose-dependent regimens contributes significantly to fatigue in cancer patients (Adams-Campbell et al., 2023; Thambiraj et al., 2022). 3) Complications: Anemia, infection, thyroid dysfunction, electrolyte imbalances, etc., arising from the tumor or its treatment, can also cause and worsen fatigue (Knefel et al., 2023). 4) Disruptions in the hypothalamus-pituitary-adrenal axis have been linked to the development of fatigue associated with tumor therapy (LN et al., 2015). 5) Psychological factors: Diagnosis, treatment, and anxieties related to prognosis, functional loss, and financial burdens can lead to insomnia, depression, tension, irritability, fear, emotional distress, sadness, and other mental and psychological issues (Chartogne et al., 2021). These mental and psychological factors can further promote and exacerbate fatigue, creating a potentially cyclical relationship. Based on this comprehensive analysis, this study has chosen to evaluate the impact of interventions on general living conditions, anti-fatigue effects, and anti-tumor activity.

General health parameters, including body weight, activity level, fur condition, and food intake, provide objective indicators of the quality of life in mice. In this study, mice in the NC group exhibited a steady increase in body weight. Conversely, mice in the TC group displayed a significant decrease in body weight compared to the NC group at weeks 2 and 3 of the intervention. This weight loss was further exacerbated in the TC + PTX group. Notably, mice in the TC + PTX + ACT group showed significantly higher body weight than the TC + PTX group. Throughout the experiment, mice in the NC group remained active with smooth, well-groomed fur, demonstrating high responsiveness to food and a quick escape reflex. In contrast, mice in the four tumor-bearing groups (TC, TC + PTX, TC + PTX + ACT, and TC + ACT) progressively displayed signs of hair loss, reduced activity, lethargy, and decreased food intake. By the experiment’s conclusion, the overall health of the tumor-bearing groups, ranked from poorest to relatively best, were TC + PTX, TC, TC + PTX + ACT, and TC + ACT. These findings suggest that the combined effects of tumor growth and its impact on feeding, digestion, and absorption in mice ultimately lead to weight loss. This aligns with observations reported in other studies, where nutritional deficiencies and anorexia are identified as potential contributors to weight loss in cancer patients. Additionally, the potential gastrointestinal toxicity and side effects of chemotherapeutic drugs, particularly when administered intraperitoneally, may exacerbate weight loss in mice. Importantly, the results demonstrated that ACT could significantly improve weight loss caused by the tumor itself and offer some degree of improvement in the negative effects of the tumor and its treatment on mental state, activity level, food intake, and fur condition.

Besides, exhaustive swimming time analysis revealed a significant decrease in the TC group compared to the NC group, indicating physical fatigue in colorectal cancer mice. Similarly, the tail suspension test demonstrated a significantly longer immobility time in the TC group than in the NC group. Notably, mice in the TC + ACT and TC + PTX + ACT groups displayed significantly shorter immobility times compared to the TC and TC + PTX groups, respectively. These findings suggest a propensity for physical fatigue in tumor-bearing mice. Furthermore, the open-field test results revealed significantly lower total exercise distance and peripheral movement distance in the TC group compared to the NC group. This can be interpreted as indicative of mental fatigue in mice from the TC group. Collectively, the results from the exhaustive swimming time, tail suspension test, and open field experiment provide strong evidence of both physical and mental fatigue in colon cancer mice, validating the establishment of a robust colon cancer mouse model.

Given that tumor presence is a fundamental factor in CRF development, this study also assessed tumor growth. The results demonstrated a significant decrease in tumor weight in the TC + PTX and TC + PTX + ACT groups compared to the TC group. Notably, the TC + PTX + ACT group exhibited the highest tumor inhibition rate. These findings suggest that ACT can enhance the anti-tumor efficacy of the chemotherapeutic drug PTX in colon cancer mice, potentially by hindering tumor growth.

Overall, this study demonstrates that ACT administration significantly alleviates fatigue in colon cancer mice, a condition arising from both the tumor and the chemotherapeutic drug PTX. These findings suggest a potential role for ACT in mitigating CRF associated with tumors. The observed improvement in fatigue may be attributed to a combination of factors, including the general health improvement in tumor-bearing mice, the enhanced efficacy of the chemotherapeutic drug PTX, and a potential reduction in depressive symptoms.

Given that CRF is a subjective experience readily influenced by various factors, diagnosing and evaluating it in its early stages proves challenging. However, significant alterations in energy, lipid, protein, and amino acid metabolism may occur during this early phase. Metabonomics, a field that directly assesses the biochemical state of tissues, offers a sensitive approach to characterizing these physiological and pathological changes. Consequently, metabonomics holds great promise for the early diagnosis and prevention of complex diseases (Martin et al., 2017).

This study aimed to identify specific plasma metabolites associated with different stages of cachexia in tumor-bearing mice and elucidate the mechanisms underlying cachexia development and its remission with ACT and PTX treatment. We compared the plasma metabolite profiles of five groups: NC, TC, TC + PTX, TC + ACT, and TC + PTX + ACT. The analysis revealed specific alterations in metabolic pathways related to the biosynthesis of branched-chain amino acids (valine, leucine, isoleucine), ubiquinone and other terpenoid-phenylenediones, tyrosine and tryptophan metabolism, topiramate and pyridine alkaloid biosynthesis, toluene degradation, taurine and hypotaurine metabolism, steroid hormone biosynthesis, secondary bile acid biosynthesis, pyrimidine metabolism, thiamine metabolism, purine metabolism, propionic acid metabolism, porphyrin and chlorophyll metabolism, and ketone body synthesis and degradation.

Compared to the control group, the tumor-bearing group displayed elevated blood levels of dl-lactic acid, fumaric acid, acetyl L-carnitine, and testosterone. Conversely, citric acid, pantothenic acid, cholic acid, and d-pantothenic acid levels decreased. Notably, the increase in acetyl-L-carnitine, an ester of L-carnitine promoting fat burning and cellular protection, suggests enhanced fat catabolism in the skeletal muscles of TC mice, contributing to muscle atrophy and fatigue. Furthermore, elevated lactic acid levels might be linked to altered fat metabolism. Studies have established a link between overweight/obesity and colon cancer, along with its influence on tumor growth and metastasis. This aligns with the observed decrease in colon cancer mice of metabolites associated with fatty acid metabolism, including lauric acid, isovalerate, and nerve acid. These findings suggest a potential upregulation of anaerobic respiration in colon cancer, leading to increased lactic acid production. Consequently, high lactic acid levels may be a contributing factor in the pathogenesis of colon cancer in this murine model.

In comparison to the tumor-bearing control group, the TC + PTX group exhibited elevated levels of several plasma metabolites, including cis and trans aconitic acid, quinolinic acid, citric acid, glycine, various fatty acids (myristic, palmitic, linoleic, oleic, arachidonic, and a 22-carbon hexaenoic acid), uric acid, and indole. Conversely, the levels of cholic acid, β-mouse cholic acid, deoxycholic acid, histamine, and cortisol decreased in the TC + PTX group. Paclitaxel is a widely recognized anti-microtubule drug that exerts its anti-cancer effects by promoting tubulin polymerization, inhibiting depolymerization, maintaining microtubule stability, and suppressing cell mitosis (Hashemi et al., 2023). Despite its efficacy in tumor treatment, PTX is known to cause neurotoxicity and muscle/joint pain, potentially contributing to chemotherapy-related fatigue.

The TC + ACT group exhibited elevated plasma metabolites like trans-aconitate, citric acid, 3-coumaric acid, ephedrine, thymine, cytosine, and panthenol-9 compared to the TC group. Conversely, levels of dl-lactic acid, acetyl L-carnitine, and taurocholate, among other metabolites, decreased. Notably, thymine and cytosine are pyrimidine bases, essential components of deoxyribonucleic acid (DNA). They can combine with deoxyribose to form deoxyribonucleosides, including thymidine. Modified forms of thymidine, such as trifluorothymidine (FTT), serve as anti-nucleic acid metabolic anti-tumor drugs. These drugs have been shown to alleviate CRF in tumor-bearing mice.

Plasma metabolite levels in the TC + PTX + ACT group differed significantly from those in the TC + PTX group. Indole-3-acetic acid and pantothenol-9 exhibited increases, whereas hippuric acid, gentian acid, adenine, indole, arachidonic acid, and protoporphyrin-9 showed decreases in the combined treatment group. Our study has revealed that the incorporation of ACT into PTX treatment appears to influence the plasma metabolite profile in tumor-bearing mice, which had previously exhibited alterations attributed to chemotherapy. While these observations are intriguing, it is important to acknowledge that they do not conclusively establish a causal relationship with the adverse effects associated with PTX. We cautiously entertain the possibility that ACT might contribute to a modulation of metabolites and proteins related to inflammatory and energy metabolic pathways, which could potentially ameliorate some of the side effects of PTX. However, this notion remains to be substantiated, and we advocate for further investigation through rigorous experimental and clinical studies to explore this possibility more thoroughly. This protective effect could potentially counteract the exacerbation of CRF associated with PTX alone.

Our investigation into the metabolite-protein relationship identified several key enzymes that could account for ACT’s anti-CRF effect to some extent. The hub proteins in both groups were primarily associated with specific functionalities: the CPY family involved in endoplasmic reticulum and mitochondrial drug metabolism, the solute carrier family on the cell membrane, the ryanodine receptor family, and proteins related to inflammation and energy metabolism (Werk and Cascorbi, 2014). Studies have shown that RYR2 (Waddell et al., 2023), a member of the ryanodine receptor family, has been linked to the development and progression of various cancers, including lung, liver, and colon cancer. It contributes to tumor cell proliferation, apoptosis, and migration, playing a crucial role in tumorigenesis and metastasis. Based on these findings, we propose that ACT may exert its anti-cachexia effects through the regulation of inflammatory response and energy metabolism via multiple targets, including CYP3A4, CYP19A1, CYP2E1, TNF, BCL-2, RYR2, and ATP2A1. Further investigation into the interactions between these proteins and ACT is warranted to inform future drug discovery and development efforts. BCL2 immobilized on COOH chip can bind ACT with an affinity constant of 88.1 µM as determined in a SPR assay. CYP3A4 immobilized on COOH chip can bind ACT with an affinity constant of 341 µM as determined in a SPR assay. From the results of SPR experiments, BCL has a better affinity with ACT. In light of these findings, we would like to underscore that, while our data provide intriguing insights, they are not yet sufficient to conclusively demonstrate that ACT can target both BCL2 and CYP3A4 simultaneously. It is with this in mind that we recognize the need for further, more nuanced studies to unravel the intricate mechanisms of action and pinpoint the specific targets of ACT with greater certainty.

This study represents a preliminary investigation into the effects of ACT on CRF. However, several limitations warrant further exploration. Firstly, identifying differential metabolites requires validation through spiking with authenticated standards. Secondly, additional studies utilizing molecular biology techniques are needed to confirm the proposed mechanisms. Ultimately, animal model fatigue metrics may not accurately reflect human conditions. To address these limitations, future research should focus on confirming the identity of key differential metabolites using authenticated standards and investigating the influence of ACT on the expression and activity of key proteins. Moreover, enhancing animal model development and refining assessment criteria are essential. These experiments could identify the target protein(s) responsible for the observed effects.

## 5 Conclusion

In conclusion, our study demonstrates that late-stage tumor cachexia in mice induces pronounced CRF and significant alterations in their plasma metabolite profiles. These differentially regulated metabolites primarily belong to tyrosine, tryptophan, pyrimidine, and purine metabolism pathways. Importantly, ACT supplementation derived from *C. tubulosa* effectively ameliorated or reversed many of these changes. Network analysis based on differential metabolites and their associated proteins revealed key proteins potentially involved in ACT’s anti-cachexia effects. To our knowledge, this is the first investigation to employ metabolomics to elucidate the anti-cachexia properties of ACT from *C. tubulosa*. This study not only deepens our understanding of the molecular mechanisms underlying ACT’s protective effects against CRF but also offers valuable insights into the potential therapeutic application of *C. tubulosa*.

## Data Availability

The metabolomics data presented in the study are deposited in the Metabolights repository, accession number MTBLS12097.
