# Supplementary material for: Effects of acteoside from Cistanche tubulosa on the plasma metabolome of cancer-related fatigue mice inoculated with colon cancer cells
Source: Front Pharmacol. 2025 Jan 13;15:1370264. doi: 10.3389/fphar.2024.1370264 (PMC11769790; doi:10.3389/fphar.2024.1370264)
Supplement: Supplementary file 1 [file Image1.pdf]

Supplementary Material

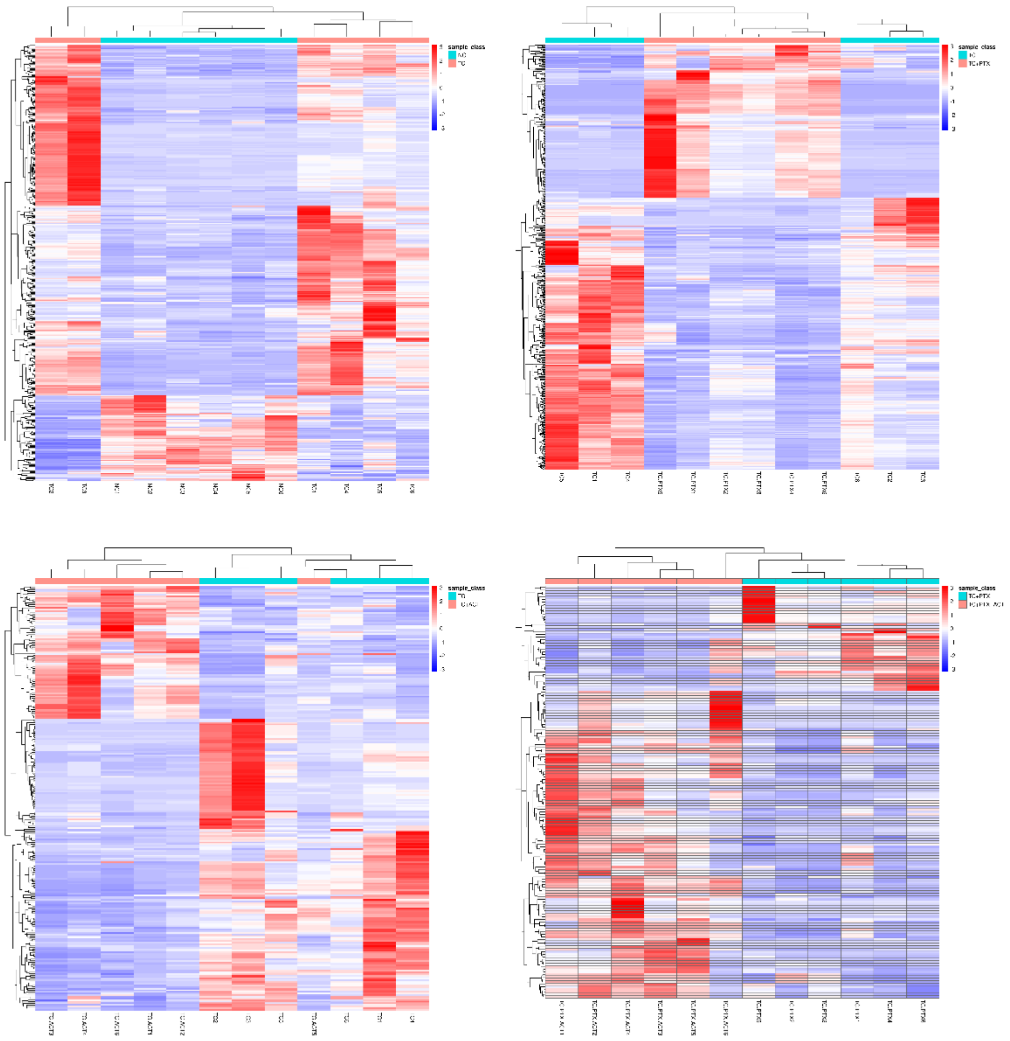

Fig. 1. A heatmap analysis with hierarchical clustering of all the groups

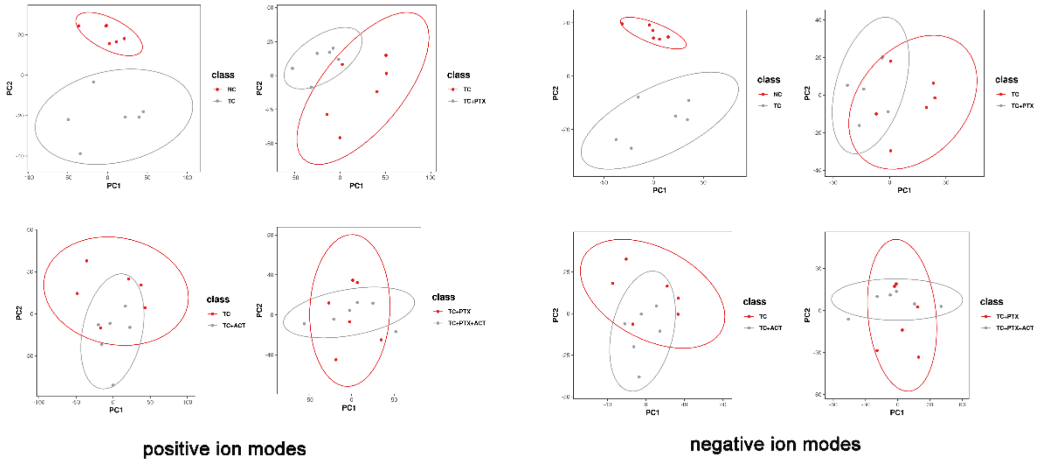

Fig. 2. The PCA score scatter plots (2D) showing clustering of plasma metabolites detected in ESI- mode (POS) and ESI + mode (NEG).

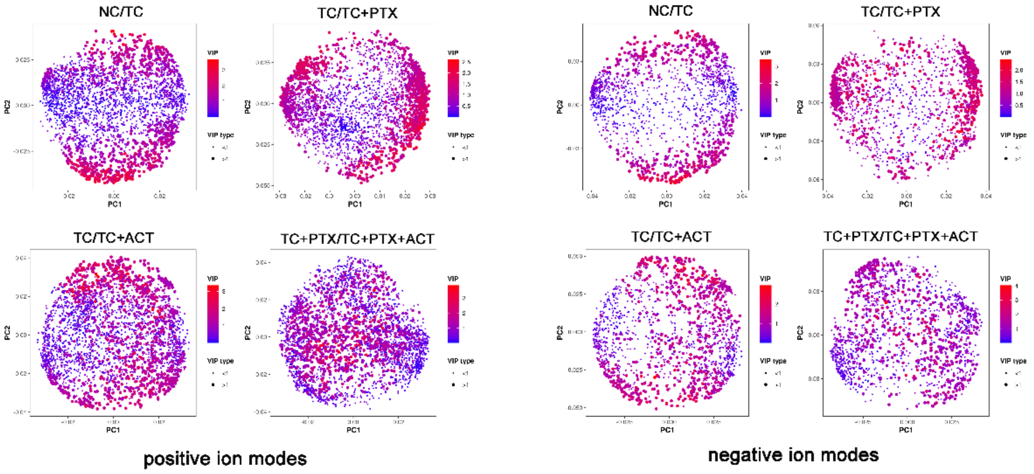

Fig. 3. The VIP metabolites PLS-DA plots showing clustering of plasma metabolites detected in ESI- mode (POS) and ESI + mode (NEG).
